# The Use of Electroactive Halophilic Bacteria for Improvements and Advancements in Environmental High Saline Biosensing

**DOI:** 10.3390/bios11020048

**Published:** 2021-02-12

**Authors:** Erin M. Gaffney, Olja Simoska, Shelley D. Minteer

**Affiliations:** Department of Chemistry, University of Utah, Salt Lake City, UT 84112, USA; erin.gaffney@utah.edu (E.M.G.); olja.simoska@utah.edu (O.S.)

**Keywords:** halophilic bacteria, microbial biosensing, microbial electrochemistry

## Abstract

Halophilic bacteria are remarkable organisms that have evolved strategies to survive in high saline concentrations. These bacteria offer many advances for microbial-based biotechnologies and are commonly used for industrial processes such as compatible solute synthesis, biofuel production, and other microbial processes that occur in high saline environments. Using halophilic bacteria in electrochemical systems offers enhanced stability and applications in extreme environments where common electroactive microorganisms would not survive. Incorporating halophilic bacteria into microbial fuel cells has become of particular interest for renewable energy generation and self-powered biosensing since many wastewaters can contain fluctuating and high saline concentrations. In this perspective, we highlight the evolutionary mechanisms of halophilic microorganisms, review their application in microbial electrochemical sensing, and offer future perspectives and directions in using halophilic electroactive microorganisms for high saline biosensing.

## 1. Introduction

**Figure 1:**
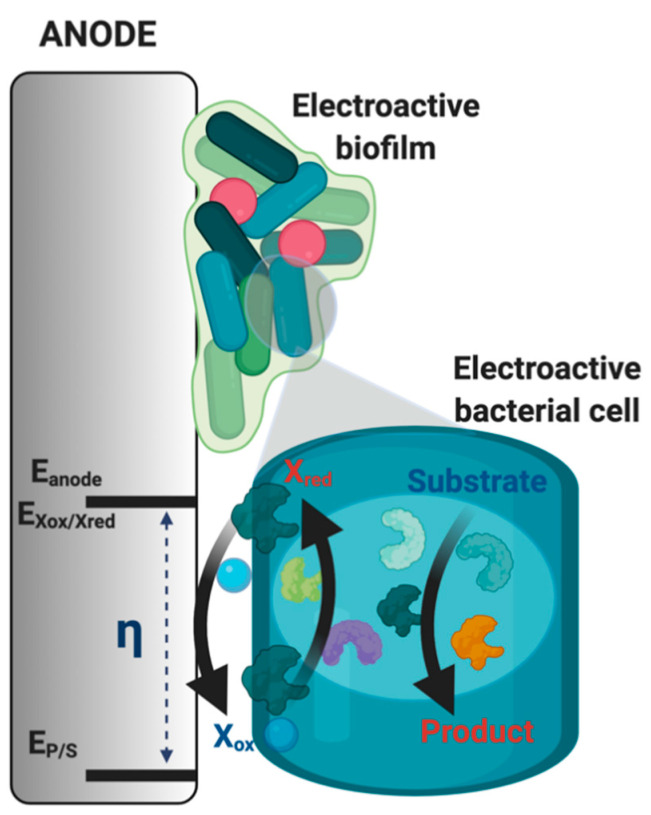
Pairing the microbial metabolism to an electrode surface (here, anode). Made with BioRender.com. As bacterial cells catalyze an intracellular redox reaction, generically represented as substrate to product, electrons flow to an eventual terminal electron acceptor such as redox-active protein on the cell surface or a biomolecule (X_ox_ going to X_red_) which is then re-oxidized at the electrode surface. Therefore, the potential of the anode (E_anode_) will be determined by the electrochemical potential of the terminal electron transfer reaction (E_Xox/Xred_). The overpotential (η) will be determined by the difference between the electrochemical potential of the metabolic redox reaction initiating electron transfer (E_P/S_) and the electrochemical potential of the reaction occurring at the anode surface.

Bioelectrochemical systems have become of great interest to the electrochemistry community for exploring avenues of renewable energy generation, biosensing systems, and bioelectrosynthesis [1,2,3]. Specifically, microbial electrochemical systems are fit for long-term environmental deployment suitable for wastewater management and monitoring applications [4,5,6]. Among the many types of microbial electrochemical systems, the technology used most often for biosensing is the microbial fuel cell since the substrate of the bacterial cells is commonly the driver of the current output. Therefore, the current output can be related to the substrate concentration in a variety of wastewaters providing a self-powered sensor [7,8,9,10]. The long-term stability stems from the ability of microorganisms to continually reproduce, allowing for a near-infinite bioelectrode where the biocatalysts are being regenerated [11]. The main challenge is identifying microbes capable of extracellular electron transfer (EET), which allows for interfacing bacterial cells with an electrode surface capable of producing an electrochemical response (e.g., current) [12,13,14,15]. Such bacterial strains can be found by harvesting in environments ripe for the evolution of bacterial strains with EET. These types of environments include areas with a lack of soluble terminal electron acceptors such as oxygen or inorganic salts. These environments create a need for the ability to solubilize insoluble electron donors outside of the cell, such as iron or sulfur oxides and inorganic metals, among other electron donor compounds [12,13]. Pairing the phenomena of EET to an electrode, generalized in Figure 1, involves utilizing the metabolic reaction of a biological substrate to a product with electron transfer steps to an electroactive molecule which is either on the cellular surface or can diffuse across the cell membrane. The terminal electron transfer step determines the potential of the anode (E_anode_ in Figure 1), and the energetics of the reaction of the substrate to the product determines the potential of the metabolic reaction (E_P/S_ in Figure 1), which creates the inherent overpotential (Figure 1) in microbial systems. This overpotential will vary in the electroactive species used in the microbial electrochemical system and the electron transfer mechanisms at play, which can be extremely complicated [16]. Many approaches are focused on studying these phenomena, including bioinformatics and computational modeling [17,18].

Many bacterial strains have been characterized and isolated for EET, with a few model organisms that have been intensely studied, including *Geobacter sulfurreducens* and *Shewanella oneidensis* [19,20,21,22]. Even with the advances of characterizing many strains capable of this feat, few organisms have been identified which are extremophilic and have the ability to perform EET [23]. Such organisms are of great interest for environmental applications of microbial electrochemical systems due to the creation of robust bioanodes capable of survival and performance in a dynamic range of environments [23,24]. Specifically, chemical environments of high saline are of interest for the development of water treatment and biosensing for contaminants, since these waters account for 5% of the total world’s liquids and result from a variety of industrial processes [25]. This perspective will discuss the phenomena of halotolerance and the environments which are of particular interest in incorporating halotolerant bacteria in microbial electrochemical biosensors. Future directions and opportunities offered by the study will also be discussed, mainly focusing on the applications and engineering of electroactive halophilic bacterial driven microbial electrochemical biosensors.

## 2. Electrifying Halophilic Bacteria

**Figure 2:**
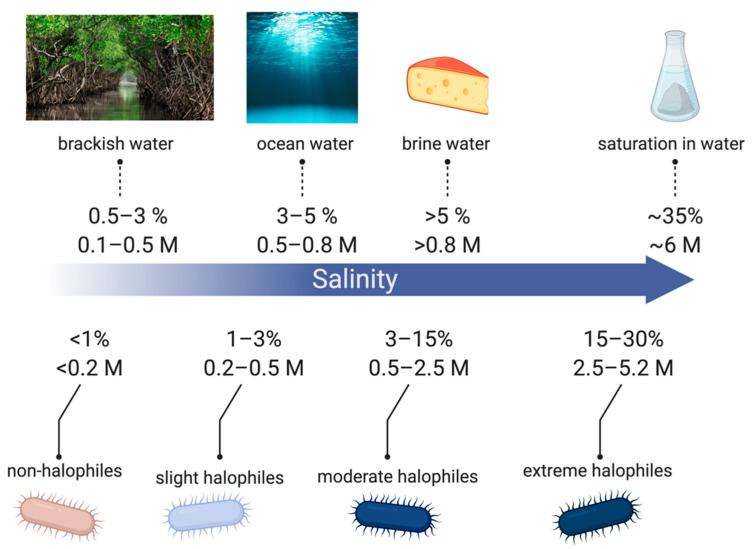
Classification of halophiles based on different levels of salt concentrations. Made using BioRender.com.

Halotolerant strains are extremely diverse among similar environments with Gram-negative and Gram-positive physiologies [26]. Common genera include Gram-negative *Halomonas*, *Pseudomonas*, *Flavobacterium*, *Halovibrio*, *Deleya*, *Chromobacterium*, *Desulfuromonas*, *Marionbacter*, and Gram-positive *Halobacillus*, *Salinicoccus*, *Nesterenkonia*, *Marinococcus*, and *Tatragenococcus* [24,26,27]. Halophilic bacteria are classified from slight to extreme halophiles depending on the salt concentrations they are capable of surviving in, as shown in Figure 2. Halophiles exist in the full range of salinity from low concentrations to salt saturation in aqueous solutions (~6 M) [28].

### 2.1. Mechanisms for Saline Tolerance

Halophilic bacteria utilize two mechanisms for halotolerance known as the salt-out and the salt-in strategies. The salt-out strategy involves the uptake or synthesis of osmoprotectants. Osmoprotectants include small molecules, such as ectoines, amino acids, sugars, and betaines, and accumulate in the cytoplasm to protect the cells from lysis caused by an imbalance of osmotic pressure [27]. The salt-in strategy used by species such as *Salinibacter ruber*, involves the accumulation of counterions in the cytoplasm through an influx of potassium ions [27]. The latter mechanism requires structural differences in biomolecules for function in high ion concentrations. However, the former is usually most common since osmoprotectants are also capable of stabilizing biomolecules in high ion concentrations. Therefore, it does not require significant structural changes [27]. Structural differences found in the potassium influx osmoregulation mechanisms include a general increase in acidic content and charged amino acids on the protein surface, and in particular, an increase of glutamic acid due to its ability to bind water molecules better than any other amino acid [27,29]. These modifications allow for the increased integration of water in the protein solvent shell and result in flexible protein structures that would otherwise be inaccessible due to the high ionic strength [30,31].

### 2.2. Halophilic Bacteria in Biotechnology

Halophilic bacteria have a wide range of use in biotechnology and are actively being explored for many applications. One of the early uses for halophilic bacteria has been the production of solar salt from seawaters and fermentation of traditional fermented foods [32]. Due to the mechanisms described in the previous subsection, moderately halophilic bacteria are often used for the bioproduction and synthesis of compatible solutes ectoine and β-carotene for enzyme stabilizing chemicals [32]. In addition to these compatible solutes, there has been recent interest with more complex molecules such as bioplastics polyhydroxyalkanoates (PHAs) and biosurfactants for enhanced microbial oil recovery [33,34], as well as molecules that could be of interest in biomedicine for antimicrobial and anticancerous properties [35]. Halophiles have also been demonstrated for biofuel production, including bioethanol, biobutanol, biodiesel, and biogas, from organic substrates and biomass due to their ability to survive in high saline concentrations which are common in a variety of industrial production processes [36]. Enzymes are commonly isolated from extreme halophiles due to their increased activity in high saline, as described above [30,31]. Halophilic enzymes, such as hydrolases, amylases, and isomerases, have been isolated from halophilic bacteria for their use in high salt solvents and biotechnological applications [32,33,34], as well as a bacteriorhodopsin proton pump, has been isolated from *halobacterium* for use in optoelectronic devices and some photochemical applications [32].

### 2.3. Electroactive Halophilic Bacteria

Halophilic bacteria have been shown to be electroactive, and in general, electroactivity is seen broadly amongst all families of bacteria [12,23]. Additionally, there have been strategies to engineer systems with non-halophilic bacteria to become salt tolerant, as well as to engineer non-electroactive to become electroactive, opening up many opportunities to create electroactive halophilic bacteria, which is discussed in detail in the future directions section [37,38,39,40,41]. Electroactive halophilic bacteria have been used in some pioneering studies. However, it is of great interest to identify and characterize more electroactive halophilic microorganisms for understanding and their future application as biocatalysts in extreme environments such as saline conditions [16,23,24].

## 3. Microbial Electrochemical Biosensing in High Saline

**Figure 3:**
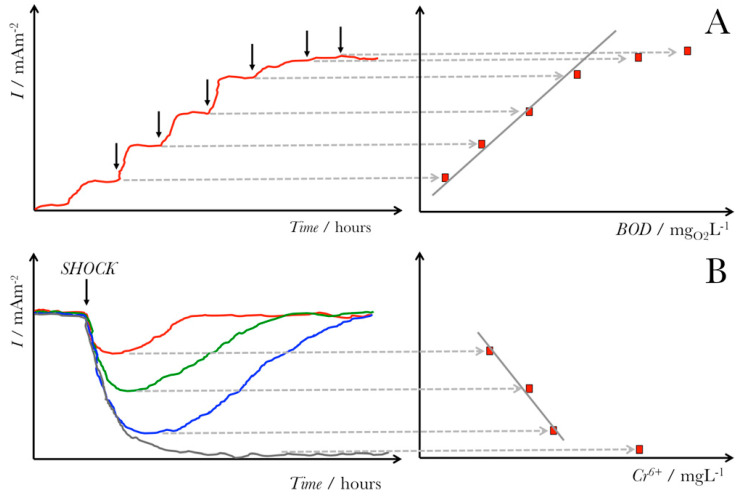
General scheme for microbial electrochemical sensors. Panel (**A**) shows how a microbial electrochemical sensor would work to sense the organic content of a solution, such as the biological oxygen demand (BOD), using the current generation. Panel (**B**) shows how a microbial electrochemical biosensor would sense a toxic contaminant such as chromium (V) in a solution based on the current decrease from the inhibition of microbial metabolic current generation, limited by the maximum concentration of Cr^5+^ that would produce an irrecoverable current drop from the death of bacterial cells. Reproduced with permission from M. Grattieri and S.D. Minteer. Self-Powered Biosensors. ACS Sens. 2018, 3, 44–53 [9]. Copyright 2018 ACS.

Although halophiles are used in numerous biotechnological applications [26,32,33], their use in microbial electrochemical technologies remains limited and is still being investigated [42,43,44,45]. Halophilic bacteria offer a promise as microorganisms in microbial electrochemical systems as their use allows for operation under extreme, harsh conditions, including high pH and elevated salinity levels [23]. Specifically, the high salinity conditions have various advantages for MESs, including (1) improved current densities, (2) decreased internal resistance, and (3) enhanced proton transfer mechanisms across the ion exchange membranes [25]. Among the possible applications of halophilic microbes is their use to design and develop molecular sensors [32] and microbial-based biosensors [24,46]. For instance, halophilic bacteria can act as biosensors for the detection of low amounts of organic carbon [24] and real-time monitoring water and toxicity [46,47]. This general concept of using microbial electrochemical biosensors for sensing organic content, such as biological oxygen demand (BOD) or toxic contaminants, is shown in Figure 3. The ability of halophilic bacterial strains to adapt and survive under extreme conditions (e.g., high alkalinity, salinity, and temperature) offers a promising sensing perspective for water quality monitoring in industrial wastewaters [48].

### 3.1. Microbial-Based Sensing in High Saline

Microbial electrochemical biosensors offer various advantages, including simplistic designs, low-cost, versatility, and selectivity towards a variety of target analytes [49]. Additionally, these biosensors offer a means for real-time, quantitative monitoring [46,50]. However, microbial electrochemical biosensors face various challenges, including limited selectivity, low detection limits, and potential contamination with other bacterial strains [46,51], which limit their real-world applications. Moreover, microbial biosensors have partial durability (e.g., several hours to a few days) [46,52,53]. Consequently, long-term real-time monitoring under harsh conditions remains limited. In this section, we provide an overview of the performance and challenges of electrochemical-based sensing in high saline environments.

For instance, Kretzschmar et al. reported a microbial electrochemical biosensor using *Geobacter* sp.-dominated biofilms on electrodes for the detection of volatile fatty acids [8]. Using this amperometry-based biosensor, the researchers followed the profiles of acetate concentrations in the anaerobic digestion (AD) course. The authors examined high salt concentrations as a potential inhibitor to biofilm growth during the AD process. Their results showed that the *Geobacter* sp. biofilms acting as the biosensor recognition element are not prone to high salinity levels, thereby having no impact on the sensor activity. A study by Hernández-Sánchez et al. reported the design of a whole-cell biosensor for the detection of monocyclic aromatic compounds [54]. This biosensor based on *Alcanivorax borkumensis* SK2 was reported to have a high tolerance towards salinity, demonstrating good performance for the detection of low-concentration pollutants in seawater samples. On the other hand, in a very recent study, Chung et al. showed a continuous closed-circuit operating microbial electrochemical cell-based biosensor for the fast detection of a naphthenic acid compound in water samples [55]. Their results revealed that the biosensors would be sensitive to both high salinity levels and temperature variations since an increase in salinity levels resulted in amplified transient peak currents from the biosensors. However, careful calibration allowed for biosensor measurements of the naphthenic acid compound. These results could potentially suggest that there is an unknown and unfavorable metabolic condition when the electrochemically active bacteria are exposed to high-salt levels, impacting the overall biosensor performance. While high salinity conditions can result in reduced internal resistance in microbial electrochemical cells [25,56], future research needs to carefully examine and study the effects in developing biosensing platforms for application in extreme, high salinity environments. In the context of whole-cell biosensors, future studies should focus on the design and manufacturing of specific and multifunctional biosensors for fast, quantitative, real-time electrochemical detection in extreme, harsh settings where there is high salinity, as well as high acidity, extreme temperatures, and toxic substances. Specifically, the utilization of numerous halophilic bacteria as host microorganisms should be carefully investigated in the design and development of microbial-based biosensors and their application for extreme environmental sensing and analysis.

**Figure 4:**
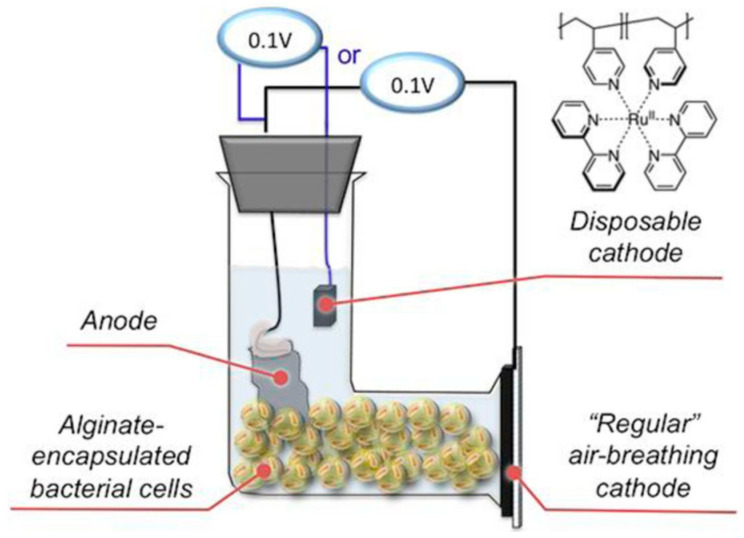
Self-powered hypersaline microbial electrochemical biosensor. Alginate encapsulated *Salinivibrio* sp. EAGSL cells were added to a single-chamber microbial fuel cell with a disposable ruthenium modified ([Ru(bpy)_2_(PVP)_5_]•Cl_2_ (PVP-Ru(bpy)_2_Cl_2_)) cathode for increased sensitivity. Reproduced with permission from M. Grattieri, D.P. Hickey, B. Alkotaini, S.J. Robertson, and S.D. Minteer. Hypersaline Microbial Self-Powered Biosensor with Increased Sensitivity. J. Electrochem. Soc. 2018, 165, H251–H254 [42]. Copyright 2018 IOP Publishing.

Another application of microbial-based biosensors is their use for sensing BOD. The conventional method to quantify BOD requires a lengthy 5–7-day procedure, trained highly-skilled personnel, and commonly toxic powerful oxidants [46]. Consequently, microbial fuel cell (MFC)-based biosensors have been designed [42,46]. As an alternative to the standard chemical oxidation BOD sensing approach, these biosensors are based on the microbial degradation of organic matter and its conversion to an electrical current (Figure 3) [9,57]. Jiansheng et al. reported microbial biosensor fabrication based on the Clark oxygen electrode and immobilized *Bacillus licheniformis* as the biological element for sensing seawater BOD [58]. The final results from this study showed that the biosensor is stable and functional in high saline environments (until NaCl was 80 g L^−1^ in samples), resulting in measurable signals when monitoring the BOD of water. In a more recent study, Grattieri et al. have reported a hypersaline microbial self-powered biosensor with increased sensitivity for chemical oxygen demand (COD) sensing [42], as shown in Figure 4.

In their design, the researchers utilized a disposable cathode based on [Ru(bpy)_2_(PVP)_5_]•Cl_2_ (PVP-Ru(bpy)_2_Cl_2_) in an MFC-based setup for self-powered, environmentally friendly monitoring of COD, resulting in higher sensitivity (by one order of magnitude) compared to the MFC setup with an air-breathing cathode. A linear relationship between the coulombs of charge and COD was established and determined to be ~10,000 mg COD L^−1^. Additionally, as part of their self-powered biosensor design, the researchers entrapped the bacterial cells (*Salinivibrio* sp. EAGSL) in alginate-capsules. As such, this biosensor design offers a means for applications as a biosensor platform in high-salt solutions.

### 3.2. Microbial Fuel Cells in High Saline with Potential for Microbial Electrochemical Biosensing

In addition to the study presented above, several microbial fuel cells in high saline environments have been developed for the treatment of wastewater and power production from urine [42,44,45,59,60,61,62,63]. Recently reviewed, microbial fuel cells in high saline have demonstrated great potential for the monitoring and sensing of the degradation of organics in high saline water [43,46]. Microbial fuel cells can also be used to power a device for biosensing in high saline [9,43]. Additionally, electrochemical systems for degradation and monitoring of toxic compounds in high saline have been investigated and have the potential to serve as a framework for microbial electrochemical systems to replace expensive anodic and cathodic materials [64].

## 4. Future Directions and Perspectives on Utilizing Halophilic Electroactive Bacteria for Electrochemical Biosensing

### 4.1. Heavy Metal Sensing with Electroactive Halophilic Bacteria

In the last two decades, microbial whole-cell biosensors have shown great potential for use in areas of environmental monitoring and biomedical diagnostics [1,50,65]. Whole-cell biosensors offer various advantages, including good sensitivity, high selectivity, and the ability for in situ, quantitative detection. Therefore, these microbial-based biosensors have been successfully applied for food and drink analysis, environmental monitoring, biomedical diagnostics, and drug screening [1,50,65,66]. In this subsection, we provide an overview of the design of microbial electrochemical biosensors using halotolerant microbes, which have been utilized for seawater BOD and heavy-metal sensing [47].

Many hypersaline sites are naturally enriched with heavy metals [67]. While the isolation of halotolerant bacterial strains surviving under elevated metal conditions was initially motivated by the usefulness of molecular markers to elucidate genetic mechanisms in such organisms, evidence has suggested that these microbes mediate the cycling of metals in natural, saline sites [67]. Therefore, halophiles show great potential to be used for heavy-metal sensing in high-salinity environments [67]. Lee et al. described the development of a whole-cell biosensor using halophilic microbe *Halomonas elongate* strain OUT30018 for the detection of metals in high-salt environments and sites [68]. This biosensor design demonstrated high sensitivity and specificity towards Cu under high salinity conditions. In a more recent study, Cui et al. utilized a halotolerant bioreporter *Acinetobacter baylyi* Tox2 with the host *A. baylyi* strain ADP1 for the detection of cytotoxicity levels of environmental, seawater samples contaminated with heavy metals (e.g., Zn, Cu, Cd) [69]. This study demonstrated the successful use of *A. baylyi* Tox2 as a rapid and sensitive biosensor element to monitor seawater cytotoxicity. Therefore, microbial whole-cell biosensors offer a means for heavy metal detection and monitoring. The biosensing constructs in bacteria are capable of producing qualitative and quantitative outputs as a response to heavy metals [70]. Future development of microbial biosensors for multiplexed heavy-metal sensing is necessary. Additionally, laboratory-based microbial biosensors need to be applied for practical on-site monitoring and detection of heavy metals. Additionally, adapting synthetic biology approaches with alternate microbial frameworks may lead to an increase in the robustness of microbial biosensors.

Given these examples, a major future trend in microbial biosensors is to design and develop sensing platforms for applications in extreme, high saline conditions, allowing for practical detection devices. Since most normal bacteria cannot survive such extreme environments and have relatively narrow ranges of environmental tolerance [71], the selection of a microorganism that can survive under extreme, high-salt conditions is a very important aspect and focus of future studies on the development of microbial biosensors and characterization of their performance [49]. These aspects are important due to the growing need for low-cost, selective, and sensitive microbial biosensors with fast response times.

### 4.2. Engineering Systems with Electroactive Halophilic Bacteria for Microbial Electrochemistry

**Figure 5:**
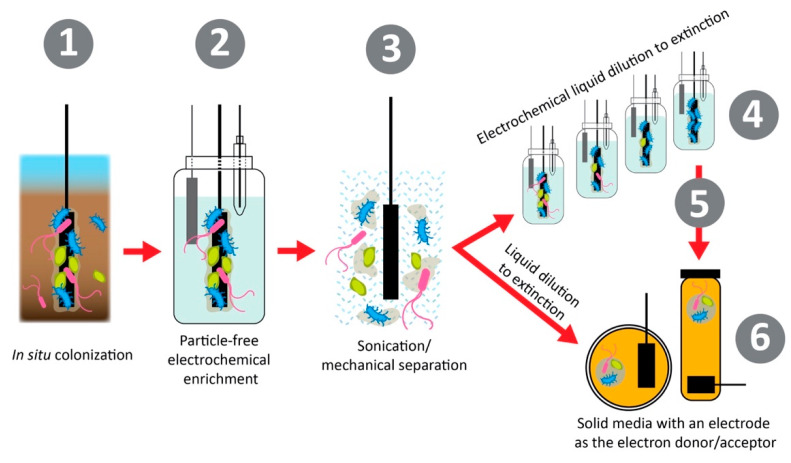
Isolating bacteria for electroactivity. First, the bacteria are colonized in situ. Then, using electrochemistry, the bacterial population is enriched around the electrode with strains capable of pairing their metabolism forming an electroactive biofilm. This biofilm is then separated from the electrode surface by sonication or other methods of mechanical separation. The microbial culture is then diluted in liquid or solid media to separate isolates from one another, eventually resulting in a single microbial isolate grown with the electrode surface as the sole electron acceptor or donor. Reproduced with permission from M.O. Yee, J. Deutzmann, A. Spormann, and A.E. Rotaru. Cultivating Electroactive Microbes, From Field to Batch. Nanotech. 2020, 31, 174,003 [13]. DOI: 10.1088/1361-6528/ab6ab5. Copyright 2020 IOP Publishing.

Isolation of both halophilic and electroactive bacteria allows for the characterization of these unique traits and physiologies, as well as a fundamental understanding of these metabolic processes that can help shape future studies for saline microbial electrochemical systems and sensing [16]. Many electroactive and halophilic strains have been identified, however, few have been extensively genetically characterized [72]. Whole-genome sequencing of halophiles allows for the investigation of the molecular mechanisms at play for halotolerance, such as ion transports or biosynthetic pathways of osmoprotectants [73,74]. Additionally, few halophiles have been isolated specifically for electroactive activity [59]. Methods for isolating electroactive bacteria are becoming more developed as the importance of keeping the ability for extracellular electron transfer (EET) can be very sensitive to electron donors in media [13]. A method to isolate and enhance EET in environmental bacterial samples is shown in Figure 5. This method focuses on first separating the bacterial cells from any insoluble or soluble particles that could act as electron donors such as iron or sulfur oxides in environmental samples. Then, the bacterial cells are diluted in liquid or solid media in a series to develop pure isolates, which are dependent on the electrode surface for electron donation (anodic respiring) or electron acceptance (cathodic respiring) [13]. The process of dilution and dependence on the electrode surface for metabolic function allows for the isolation of bacteria capable of EET, which should further be studied for the elucidation of their EET mechanisms. This framework should be applied to environmental areas of interest for microbial electrochemical systems and sensing to allow for corresponding tolerance [24].

With novel electroactive and environmental tolerant bacterial strains being studied and advances in next-generation sequencing technologies, genetic mechanisms for these traits are being found and applied in model microbial systems. Genetic engineering for electroactive halophilic bacteria has been accomplished by introducing genes for outer membrane cytochrome proteins for EET at the cellular surface and biosynthetic pathways for the synthesis of electron mediator molecules such as phenazines [41,75,76,77]. These strategies have not yet been employed in halophilic bacteria, but the advances in synthetic biology approaches demonstrate an exciting avenue for future research. Additionally, with increased knowledge of halotolerance molecular mechanisms gained from studying halophilic bacteria [73,74,78], non-halotolerant electroactive bacteria have been able to survive and generate increased currents in saline environments by the addition of compatible solutes such as glycine betaine to aid in halotolerance [37,38].

Additionally, with the advancement of knowledge of microbial electrochemical systems, biofilm formation has been found to be paramount to the success of these systems [79]. Quorum sensing is a bacterial phenomenon of small molecule communication between bacterial cells to induce community changes such as biofilm formation [80]. The bacterial mechanisms of quorum sensing can be harnessed to increase electroactive biofilm formation and thereby improve the current production in microbial electrochemical systems [81]. For the use of halophilic bacteria in microbial electrochemical systems and sensing, quorum sensing should be investigated and utilized to increase electroactive biofilm formation. Halophilic bacteria are known to produce acyl homoserine lactone autoinducers for quorum sensing and resulting biofilm formation, which provides an opportunity for the autoinducers to be used for the enhancement of current generation in saline microbial electrochemical systems and sensors [80,82]. The utilization of other autoinducers, such as quinolone-based molecules, has also been shown to increase the current responses in microbial fuel cells in high saline media using the halophilic bacterium *Halanaerobium praevalens* [83]. Quorum sensing autoinducers of biofilm formation should be further investigated for their use in improving microbial electrochemical systems with a special interest in elucidating quorum sensing pathways in halophilic bacteria.

### 4.3. Extremozymes from Halophilic Bacteria for Enzymatic Sensing in High Saline Environments

Another interesting aspect for future studies is to examine and characterize halophilic enzymes isolated from salt-loving bacteria for the design of enzymatic-based biosensors. Enzymes isolated from extremophilic bacteria, called extremozymes, have been of interest for many industrial applications in extreme conditions [84]. Genomic and structural analysis have recently established that enzymes of halophilic bacteria are negatively charged due to excessive acidic residues [23], thereby enhancing their solubility and allowing for the formation of a solvation shell to maintain the enzyme stability under high salinity conditions [85]. Such novel halophilic enzymes isolated from halotolerant microorganisms could provide enzyme stability and activity and the ability to operate as biosensing elements under extreme, harsh conditions, opening up new exciting opportunities for the design of enzymatic biosensors [86]. Finally, halophilic enzymes could also be expressed on cell surfaces via surface expression anchors, where microorganisms can serve as a support matrix for the enzymes. These approaches could result in the development of highly selective and sensitive microbial sensors with fast response times.

## 5. Conclusions

Microbial electrochemical biosensors offer a great opportunity for environmental monitoring and degradation applications. The major limitations of these technologies can be the durability and stability of the electroactive bacteria since they are the biocatalysts in these sensing systems (namely, the microorganism is the biological recognition element of the biosensor). By utilizing halophilic bacteria and their mechanisms, microbial electrochemical biosensors can be greatly enhanced to improve the overall sensor performance. The few pioneering studies in this field reviewed herein, along with this perspective, aim to point out the many exciting future directions that can be gained from these studies. These intriguing areas focused on heavy-metal sensing, extremely tolerant bacterial cells, and extremozymes will allow for the design and development of novel biosensors for extreme environmental monitoring.

## Data Availability

Data sharing not applicable.
